# Development of Neutralizing Nanobodies to the Hemagglutinin Stem Domain of Influenza A Viruses

**DOI:** 10.32607/actanaturae.11495

**Published:** 2021

**Authors:** D. V. Voronina, D. V. Shcheblyakov, I. B. Esmagambetov, A. A. Derkaev, O. Popova, D. N. Shcherbinin

**Affiliations:** FSBI “National Research Centre for Epidemiology and Microbiology named after Honorary Academician N.F. Gamaleya” of the Ministry of Health of Russia, Moscow, 123098 Russia

**Keywords:** Nanobodies®, VHH, phage, display, influenza virus

## Abstract

The influenza virus infection claims ~650,000 lives annually. Taking into account the evolving resistance of the pathogen to antiviral drugs and the waning effectiveness of vaccination among certain populations, new approaches to the treatment of influenza are needed. The current study is aimed at obtaining single-domain antibodies (Nanobodies®) to the highly conserved stem domain of influenza A virus hemagglutinin by phage display. Two high-affinity neutralizing clones of Nanobodies® with a particular specificity were selected; they ensured 100% neutralization of the H1N1 and H5N2 influenza viruses in vivo. The obtained data demonstrate that it is possible to develop highly effective VHH-based drugs for the treatment of influenza.

## INTRODUCTION

Influenza remains a serious public health risk despite the large number of vaccines and etiopathogenetic approaches to its treatment developed every year. Vaccination remains the most surefire strategy against the influenza infection to date. Antiviral drugs targeting both seasonal and pandemic influenza strains complement existing prevention strategies against the virus. However, given the increasing drug resistance, reduced vaccination efficacy in certain populations, and the short therapeutic window for the available antiviral drugs, there is an urgent need for a new type of drugs against the influenza virus infection.

Production of antibodies against highly conserved regions of viral proteins can be an effective strategy to treat influenza. Hemagglutinin (HA) is one of the major proteins of the influenza virus envelope; it forms trimers on the virion surface. HA monomers consist of two subunits: HA1 and HA2. HA contains the following spatial elements: the globular head domain, which includes the central part of the HA1 subunit, and the distal stem (stalk) domain (SD) formed by the HA2 subunit and the N- and C-terminal regions of the HA1 subunit [1]. The overwhelming majority of antibodies are directed against the highly immunogenic region around the receptor-binding site located in the globular domain, thereby simultaneously providing virus neutralization and exerting immunological pressure, leading to the emergence of escape mutants [2]. These antibodies are almost always either strain- or subtype-specific. SD is, on the other hand, less immunogenic; however, antibodies against it often recognize several HA subtypes due to its highly conserved sequence [2]. Thus, the development of anti-SD antibodies is considered a promising strategy in the pursuit of new antiviral drugs.

Currently available monoclonal antibodies (mAbs) against SD have a wide spectrum of reactivity: from binding HA subtypes within one phylogenetic group [3-10] to recognizing HA of both groups [11-21], and even exerting cross-reactivity between type A and B HA proteins [22]. Most of these antibodies recognize the conserved conformational epitopes in SD. In addition to conventional antibodies, single-domain anti-SD antibodies (nanobodies, VHH) have been obtained [23, 24]. All reported nanobodies are cross-reactive and either neutralize viruses of the same HA phylogenetic group or, as in the case of multispecific antibodies, acquire the ability to neutralize both type A and B influenza viruses.

VHH is a variable domain of heavy-chain immunoglobulins (HcAbs) found in Camelidae [25]. Despite their small size (12–15 kDa), VHHs are not inferior to conventional antibodies in affinity and specificity. Due to their unique stability in a wide temperature range, resistance to the action of various detergents and proteolytic cleavage, single-domain antibodies can be delivered in the body orally and by inhalation [26, 27]. Nanobodies are used to treat oncological, hematological, infectious, and autoimmune diseases; such drugs are either undergoing clinical trials or have been approved for use in European countries and the United States [28, 29].

In this study, we obtained a stabilized SD trimer with preserved conformational epitopes of the neutralizing antibodies and selected virus-neutralizing anti-SD VHHs by phage display. We selected two high-affinity clones exhibiting 100% neutralization of the H1N1 and H5N2 influenza viruses in a model of lethal infection in vivo. The possibility of developing highly effective VHH-based drugs for the treatment of influenza has been demonstrated.

## EXPERIMENTAL

**Biological materials**

The following highly purified preparations of recombinant proteins were used in the study: full-length HAs of the influenza A viruses H3N2 (A/Switzerland/9715293/2013) and H1N1 (A/California/04/2009) (Sino Biological, China). Restriction endonucleases, T4 DNA ligase, and alkaline phosphatase (FastAP) were obtained from NEB (USA) and Thermo Fisher Scientific (USA). The trivalent inactivated polymer-subunit influenza vaccine Grippol® plus (NPO Petrovax Pharm LLC, Russia) was used for alpaca immunization.

**HA SD synthesis**

The nucleotide sequence corresponding to the amino acid sequence of the influenza H1N1 strain SD (A/Brisbane/59/2007) (HA stem) #4900 reported by Impagliazzo A. et al. [30] was obtained from Evrogen JSC (Russia) and cloned into the pShuttle-CMV plasmid (Stratagene, USA) to obtain the pShuttle-CMV-HAstem plasmid. Next, CHO-S cells (Thermo Fisher Scientific, USA) were transiently transfected with pShuttle-CMV-HAstem using a CHOgro Expression System (Mirus Bio, USA) according to the manufacturer’s instructions. The cells were cultured in Erlenmeyer flasks at 125 rpm, 5% CO_2_, 80% humidity, and 37°C; the temperature was lowered to 32°C after 24 h, and the cells were incubated for another 10 days. Starting from day three, Cell boosts 7a (2%) and 7b (0.2%) (HyClone, USA) and 0.5% CHO Bioreactor Feed Supplement (Sigma, USA) were added once a day. After 10 days, the culture medium was clarified by centrifugation at 5,000 g. HA SD was purified by affinity chromatography on a AKTA Start Protein Purification System (Cytiva, Sweden) using 1-ml HisTrap HP columns (Cytiva, Sweden) according to the manufacturer’s instructions. Additional purification and buffer exchange for 20 mM sodium phosphate and 150 mM sodium chloride were performed on a XK 26/100 column (Cytiva) packed with 200 pg of the Superdex sorbent (Cytiva).

**Animal immunization**

An alpaca (Vicugna pacos) was immunized five times with a 14-day interval between the first and the second injection and a 10-day interval between the subsequent ones. For primary immunization, the animal was injected subcutaneously with a preparation containing 100 μg of Grippol® plus vaccine and Freund’s complete adjuvant (FCA; Sigma) mixed in a 1 : 1 ratio until a homogeneous suspension was obtained. The next four injections contained a combination of Grippol® plus and Freund’s incomplete adjuvant (FIA; Sigma). Before immunization and seven days after the fifth injection, a small amount of blood (5–10 ml) was taken from the animal as a control to determine the level of specific antibodies. One week after the last injection, 50 ml of venous blood were collected into a sterile container with lithium heparin anticoagulant. The peripheral blood mononuclear cell (PBMC) fraction was obtained using the standard protocol by centrifugation with a Ficoll solution at a density of 1.077 g/ml (PanEco, Russia).

**Phage library construction and selection of individual clones**

Messenger RNA isolation, polymerase chain reaction (PCR) of the target DNA fragments, and library construction were performed according to the standard protocols [31, 32]. The variable domains of HcAbs from the peripheral B lymphocytes of immunized alpaca were cloned into the phagemid vector pHEN1. Specific two-step PCR primers contained the SfiI and NotI restriction sites at the 5’ and 3’ end, respectively. Amplified VHH sequences were cloned into the restriction sites using endonucleases SfiI and NotI and T4 DNA ligase. Electrocompetent Escherichia coli TG1 cells were transformed with the recombinant plasmid DNA. As a result, a basic library of nanobodies, which included 3 × 10^6^ individual clones, was obtained.

Phages carrying anti-SD nanobodies were obtained after three rounds of selection (panning). A total of 5 and 1 μg of HA SD of H1N1 (A/Brisbane/59/2007) were used as an antigen in the first and next two rounds of selection. Plasmid DNA was isolated from individual selected clones, and VHHs were sequenced.

**Nanobody expression and purification**

In order to express candidate nanobodies, recombinant phagemid DNA isolated from the selected individual clones of TG1 cells was transformed into E. coli BL21 cells. Bacterial cells were grown in a liquid medium at 30°C overnight, pelleted by centrifugation, and lysed with the BugBuster Protein Extraction Reagent (Novagen, USA) according to the manufacturer’s instructions. The nanobodies were purified using TALON Superflow cobalt-charged resin (GE Healthcare Bio-Sciences AB, Sweden); the eluted fraction was dissolved in phosphate-buffered saline (PBS). The expression level was evaluated by denaturing 12% polyacrylamide gel electrophoresis (PAGE).

**Protein electrophoresis**

The proteins were separated by 12% SDS-PAGE (Bio- Rad, USA) according to Laemmli. For non-reducing non-denaturing electrophoresis, samples were mixed with a loading buffer without 2-mercaptoethanol and loaded into gel wells without preliminary heating. Precision Plus Protein™ (Bio-Rad, USA) was used as the molecular weight standard.

**Enzyme-linked immunosorbent assay (ELISA)**

To evaluate the serum levels of the antibodies in alpaca, serum samples were added to the plate wells containing either recombinant HA or recombinant SD (1 μg/ml each) immobilized in standard 0.05 M carbonate-bicarbonate buffer (pH 9.6) and then treated with IgG Goat anti-Llama IgG Heavy and Light Chain antibodies conjugated with horseradish peroxidase (HRP) (Bethyl Laboratories, USA). Library enrichment was estimated using HRP-conjugated Anti-M13 antibodies (Sino Biological, China). For the indirect analysis of the nanobodies, Rabbit Polyclonal c-Myc antibodies conjugated to HRP (Abcam, UK) were used. A 3,3’,5,5’-tetramethylbenzidine solution (Bio-Rad, USA) was used as the HRP substrate. The optical density was measured at 450 nm using a Varioskan LUX Multimode Microplate Reader (Thermo Fisher Scientific, USA).

**Surface plasmon resonance (SPR)**

The affinity and kinetics of the nanobody–antigen (HA SD) interaction were determined using a Biacore 3000 four-channel optical biosensor (GE Healthcare Bio-Sciences AB, Sweden). The recombinant SD protein (20 μg/ml solution in 10 mM acetate buffer; pH 4.5) was covalently immobilized on the surface of a CM5 sensor chip using an Amine Coupling Kit (GE Healthcare Bio-Sciences AB, Sweden). The level of immobilized ligand in the test channel of the optical biosensor was 1,800 RU.

Kinetic parameters were analyzed by injecting fivefold dilutions of nanobody samples in the concentration range of 0–267 nM through the control (without the immobilized ligand) and test channels for 3 min at a constant flow rate of 15 μL/min. HBS-EP (0.01 M HEPES, pH 7.4; 0.15 M NaCl; 3 mM EDTA; and 0.005% Surfactant P20) was used as the working buffer. The dissociation time after sample loading was 10 min. After each measurement, the chip surface was regenerated by injecting 100 mM Tris-HCl buffer (pH 1.3) for 30 s at a flow rate of 30 μL/min. All measurements were carried out at 25°C in at least two replicates.

The equilibrium dissociation and association constants (K_d_ and K_a_) and the rate constants of formation (k_on_) and decay (k_off_) of the molecular complexes were calculated using the BIAEvaluation software (GE Healthcare Bio-Sciences AB, Sweden).

**Evaluation of VHH neutralizing activity in vivo**

The studies were performed on 6-week-old female BALB/c mice weighing 18–20 g. Mouse-adapted influenza viruses H1N1 (A/Duck/mallard/Moscow/4970/2018) and H5N2 (A/Mallard duck/Pennsylvania/10218/84) kindly provided by the Laboratory of Molecular Biotechnology of the N.F. Gamaleya National Research Center for Epidemiology and Microbiology of the Ministry of Health of the Russian Federation were used to infect the animals.

The animals were divided into groups (test and control) of five animals each and infected intranasally with a mixture of 200 μg of antibody and 15 LD_50_ of virus pre-incubated at 37°C for 1 h either in a volume of 50 μL/mouse (test groups) or with the virus at a dose of 15 LD_50_ in PBS (control groups). The mice were observed for 14 days after infection; they were examined and weighed on a daily basis. Agonizing animals and animals that had lost more than 25% of their initial weight were euthanized by cervical dislocation. The neutralizing effect of the nanobodies was assessed by the survival rate and changes in the body weight of the mice.

**Statistical data analysis**

The statistical data was analyzed using the Microsoft Excel and GraphPad Prism 7 software.

## RESULTS

**Stabilized HA SD trimer**

**Fig. 1:**
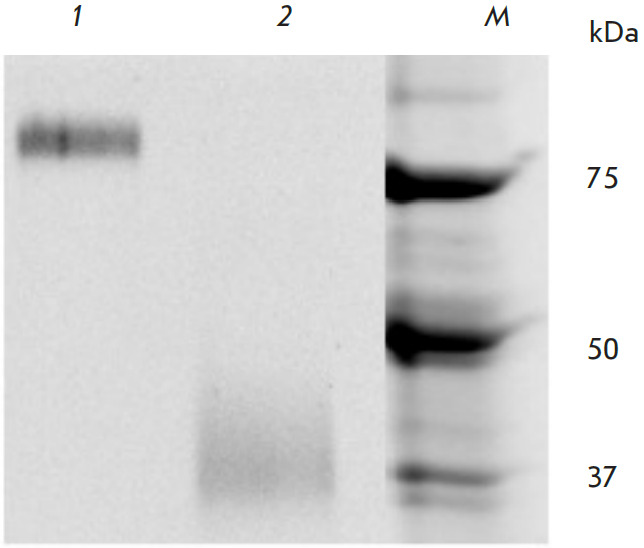
12% PAGE analysis of SD. *1 *– Non-reducing non-denaturing conditions. *2 *– Reducing denaturing conditions. *M *– molecular weight ladder

We used the amino acid sequence of the stabilized SD trimer from the H1N1 strain (A/Brisbane/59/2007) reported by Impagliazzo A. et al. [30]. In order to increase the level of HA SD expression, the HA signal peptide sequence was replaced with the signal peptide sequence of SEAP alkaline phosphatase. HA SD was produced in CHO-S cells, which provide a high level of recombinant protein expression [33]. An SD preparation of optimal purity was obtained after two purification stages: affinity chromatography and gel filtration. SD trimerization was confirmed by reducing denaturing (to visualize the monomeric structure; molecular weight, 37 kDa) and non-reducing non-denaturing (to visualize the trimeric structure; molecular weight, ~110 kDa) electrophoresis (Fig. 1).

**Production of anti-SD nanobodies**

**Fig. 2:**
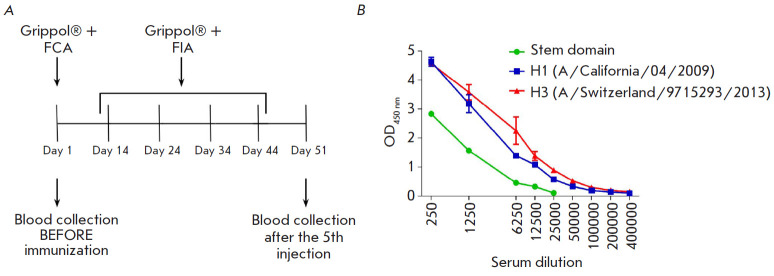
(*A*) – Schematic representation of alpaca immunization with the Grippol® plus vaccine in combination with either Freund’s complete adjuvant (FCA) or Freund’s incomplete adjuvant (FIA). (*B*) – serum levels of antibodies to SD and full-length HAs in alpaca after five immunizations

A panel of anti-SD nanobodies was obtained by immunizing alpaca (V. pacos) according to the scheme presented in Fig. 2A. On day seven after the last injection, 50 ml of blood were collected from the animal. The PBMC fraction was separated for total RNA isolation and immune library preparation. The serum was also collected to assess the induction of the humoral immune response. The serum levels of antibodies in alpaca were determined for both HA SD and recombinant HAs (Fig. 2B). The titer of anti-SD antibodies was 1 : 12,500. A high titer of anti-H1 (A/California/04/2009) and anti-H3 (A/Switzerland/9715293/2013) antibodies was revealed: 1 : 204,800 and 1 : 409,600, respectively. These results indicate a strong humoral response after five cycles of alpaca immunization with the influenza vaccine, both against full-length HAs and SD.

**Fig. 3:**
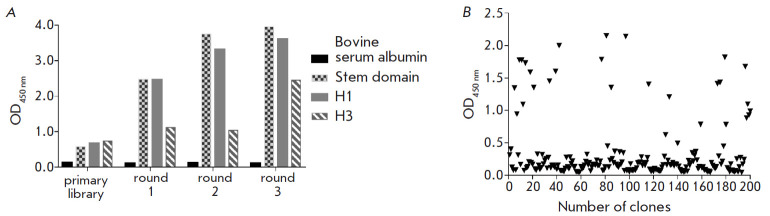
**(***A*) – Polyclonal phage ELISA: phage binding after different rounds of selection using SD and full-length HAs. (*B*) – Screening of randomly selected monoclones by phage ELISA

Construction of a nanobody library and subsequent selection by phage display were performed as described previously [34]. The library size was 3 × 10^6^ individual clones. All 30 colonies, randomly selected and analyzed by PCR for the presence of the VHH gene fragment, contained an insert. The phage library was subjected to three rounds of selection, and the results of each round were monitored by polyclonal phage ELISA (Fig. 3A). At the end of panning, 66 individual clones with an ELISA OD_450_ value above 0.25 were sequenced by Sanger (Fig. 3B). Based on the results of the CDR3 region analysis, these clones were combined into eight groups. Of these, four clones were selected for further study (B6.2, 2F2, H1.2, and G2.3) based on the specific activity in monoclonal phage ELISA and the protein expression level.

**In vitro characterization of nanobodies**

**Fig. 4:**
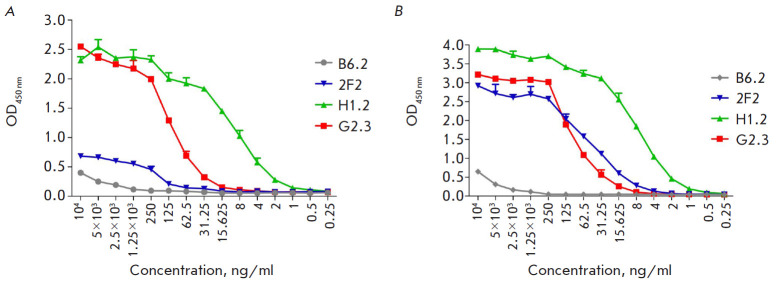
Titration of selected VHH clones by ELISA using SD (*A*) and full-length HA of the H1N1 influenza virus (A/California/04/2009) (*B*)

The specific activity of nanobodies was confirmed by indirect ELISA, and the kinetics of interaction with SD and affinity were assessed by SPR. Full-length HAs of influenza viruses H1N1 (A/California/04/2009) and H3N2 (A/Switzerland/9715293/2013), as well as HA SD (Fig. 4), were used as the antigen in ELISA; HRP-conjugated polyclonal c-Myc antibodies were used for VHH detection. Calibration curves were constructed, and EC_50_ values were determined for the clones 2F2, H1.2, and G2.3, based on the OD dependence on the antibody concentration. The EC_50_ values were 0.7, 7.4, and 13.8 nM for the interaction of SD with the clones H1.2, G2.3, and 2F2, respectively, and 0.6, 5.9, and 3.0 nM for the interaction of H1 HA with the same clones, respectively. There was no significant signal for the interaction between the antibodies and HA H3.

**Table 1:** Kinetic parameters of the interaction between VHH and SD determined by SPR

Clone	k_on_ (1/Ms)	k_off_ (1/s)	R_max_ (RU)	K_a_ (1/M)	K_d_ (M)	Chi^2^
2F2	3.95 × 10^5^	6.17 × 10^-3^	51.7	6.38 × 10^7^	1.57 × 10^-8^	1.53
H1.2	9.87 × 10^5^	3.6 × 10^-4^	139	2.74 × 10^9^	3.65 × 10^-10^	1.59
G2.3	3.68 × 10^5^	2.04 × 10^-4^	154	1.8 × 10^9^	5.54 × 10^-10^	1.39

The affinity between SD and the clones 2F2, H1.2, and G2.3 was studied by SPR using the Biacore 3000. For this, the recombinant protein was covalently immobilized on the surface of a CM5 sensor chip. Association and dissociation constants were determined by analyzing sensograms in the BIAEvaluation software. The results are shown in the Table.

**Neutralization in in vivo experiments**

**Fig. 5:**
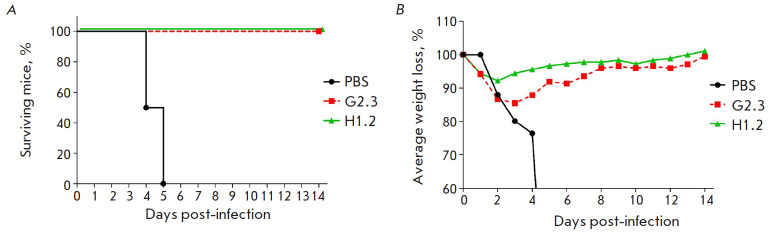
Changes in the survival rate (*A*) and body weight (*B*) of mice after intranasal infection with 15 LD_50_ of H1N1 (A/Duck: mallard/Moscow/4970/2018) pre-incubated with VHH. The differences in the survival rate between the experimental and control groups are statistically significant (*p * < 0.0005)

The neutralizing activity of nanobodies was studied using the mouse-adapted influenza virus H1N1 (A/Duck/mallard/Moscow/4970/2018). For that purpose, the mice were infected intranasally with high lethal doses (15 LD_50_) of the virulent H1N1 strain pre-incubated with either VHH H1.2 or G2.3. The animal survival rate in the test groups was 100% at 100% mouse death in the control group (Fig. 5A), which indicates effective neutralization of the H1N1 virus by both nanobodies. The neutralizing effect of the antibodies in vivo is further confirmed by a slight decrease in mouse body weight and its rapid recovery in the test groups compared to the control (Fig. 5B).

**Fig. 6:**
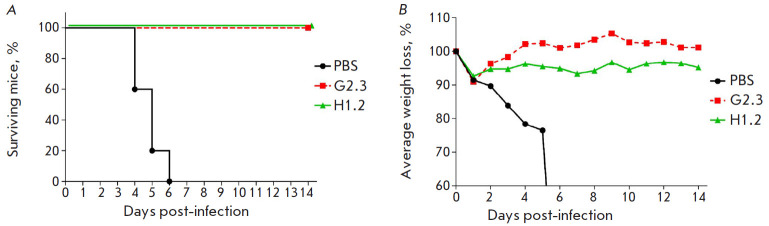
Changes in the survival rate (*A*) and body weight (*B*) of mice after intranasal infection with 15 LD_50_ of H5N2 (A/Mallard duck/Pennsylvania/10218/84) pre-incubated with VHH. The differences in the survival rate between the experimental and control groups are statistically significant (*p * < 0.0002)

An in vivo study of H5N2 (A/Mallard duck/Pennsylvania/10218/84) neutralization was carried out similarly to H1N1 neutralization. The obtained data indicate 100% neutralization of the H5N2 virus by nanobodies (Fig. 6).

## DISCUSSION

The advantages of nanobodies over conventional mAbs make them an attractive platform for the development of therapeutic agents, including antiviral agents. Because of the unique structure of its variable domain, VHHs can interact with difficult-to-reach epitopes on the viral surface [35, 36]. The smaller VHH footprint compared to conventional mAbs, which bind to larger and flatter epitopes, may constitute a greater genetic barrier for the emergence of escape mutations. In addition, the high stability and solubility of nanobodies are extremely important when creating an effective drug that can be delivered directly to the site of the infection: the lungs.

Most mAbs capable of neutralizing different subtypes of influenza viruses recognize conserved conformational epitopes in HA SD; however, they are difficult to access in a natural infection and immunization with full-length HA due to predominant exposure of variable epitopes of the HA globular domain. For this reason, what is necessary is SD with an optimal stabilized conformation, with preserved mAb-neutralizing epitopes. Impagliazzo A. et al. [30] obtained several variants of the stabilized HA SD trimer, of which #4900 can induce antibody production and provide protection against various influenza A subtypes in mice. Using the SD #4900 sequence, we obtained a preparation whose trimeric structure was confirmed by electrophoresis and that was further used to select high-affinity antibodies capable of protecting mice from various subtypes of influenza A.

We have obtained nanobodies against HA SD that potentially recognize conserved conformational epitopes and exhibit neutralizing activity against different subtypes of the influenza A virus. Four individual clones – B6.2, 2F2, H1.2, and G2.3 – were obtained after selection; they were characterized by the level of specific activity against SD and full-length HA of the subtypes H1 and H3 in indirect ELISA, affinity in SPR analysis, and in in vivo neutralization tests.

An ELISA analysis showed that the clones H1.2 and G2.3 establish the strongest interaction with SD and full-length H1 HA. A similar signal was observed for the interaction between the clone 2F2 and H1 HA; however, in the case of SD, it was lower for 2F2 compared to H1.2 and G2.3. Clone B6.2, even at high concentrations, weakly reacted with the antigens (Fig. 4). At this stage, clone B6.2, which exhibited the lowest titer in ELISA and, presumably, had the lowest affinity, was excluded from further study. According to the ELISA data, the clones we selected do not bind to H3 HA; however, many available broad-spectrum mAbs neutralize HA only within one phylogenetic group. In addition to evolutionary similarity, these groups share common conserved epitopes of cross-neutralizing antibodies in the SD hydrophobic pocket [4]. Monoclonal Abs binding this antigenic site preferentially neutralize HA of the same group and either do not neutralize the subtypes of the other one or neutralize them with less efficiency.

The K_d_ values correlate with those obtained in indirect ELISA: the lowest dissociation constants (nanomolar range) are characteristic of the antibodies H1.2 and G2.3. Despite a EC_50_ similar to those of the clones in ELISA for H1N1, 2F2 exhibited significantly lower specificity and affinity for SD. The in vitro experiments allowed us to select the clones H1.2 and G2.3 with the highest affinity (K_d_ 3.65 × 10-10 and 5.54 × 10^-10^ M, respectively) for in vivo characterization of antibodies.

Validation of the neutralizing activity of the nanobodies H1.2 and G2.3 against a lethal dose of a mouse-adapted H1N1 virus (A/Duck: mallard/Moscow/4970/2018) ensured 100% protection to mouse.

Based on the phylogenetic proximity of the viruses of the HA subtypes H1 and H5 and the conservation of their SD amino acid sequence, we assumed that the selected antibodies can bind and neutralize influenza virus strains with the HA subtype H5 in vivo [37]. The adapted H5N2 influenza virus (A/Mallard duck/Pennsylvania/10218/84) was selected for animal experiments. The HA SD sequences of the influenza viruses used in our animal experiments were first analyzed in the Geneious Prime software. There was an 83% homology between the sequences. Both nanobodies H1.2 and G2.3 were shown to display 100% neutralizing activity against an influenza virus with the HA subtype H5. This may be due to the high degree of homology between the HA SDs of the selected strains and, as a consequence, preservation of the VHH binding sites in G2.3 and H1.2.

The new single-domain antibodies we obtained have an extremely high affinity, they bind and effectively neutralize viruses with the HA subtypes H1 and H5; however, it is possible to further increase the binding and neutralizing ability of VHH by creating bivalent and bispecific constructs with the Fc fragment. Thus, affinity is enhanced thanks to the increased avidity, and effector functions such as antibody-dependent cellular cytotoxicity, antibody-dependent phagocytosis, and antibody-mediated complement-dependent cytotoxicity are gained; all of them are critical in terminating an influenza infection.

Antibodies capable of neutralizing HA of the first phylogenetic group are highly important, since this group also includes viruses with pandemic potential. The VHHs obtained by us ensured neutralizing activity against viruses carrying the HA subtypes H1 and H5. The presence/absence of neutralizing activity against other influenza strains will be the subject of further study.

## CONCLUSIONS

We have obtained a stabilized SD trimer of H1 HA (A/Brisbane/59/2007) containing conformational mAb epitopes with a broad spectrum of neutralizing activity.

The new nanobodies H1.2 and G2.3 identified by us specifically bind SD with dissociation constants exceeding those of many known monomeric VHHs and also effectively neutralize the influenza viruses H1N1 and H5N2 belonging to the first phylogenetic group of HA. We obtained constructs of these antibodies with the Fc fragment which will be used for in vivo study of protection against various influenza A virus strains.

## Abbreviations

HA: hemagglutinin
SD: stem domain of HA
HcAb: camelid heavy-chain-only antibody
VHH: variable domain of a HcAb
PCR: polymerase chain reaction
ELISA: enzyme-linked immunosorbent assay
PBMC: peripheral blood mononuclear cell
PAGE: polyacrylamide gel electrophoresis
HRP: horseradish peroxidase
EC50: half maximal effective concentration
SPR: surface plasmon resonance
LD50: median lethal dose
mAb: monoclonal antibody

## References

[1] Gamblin S.J., Skehel J.J. (2010). J. Biol. Chem..

[2] Krammer F., Palese P. (2015). Nat. Rev. Drug Discov..

[3] Ekiert D.C., Friesen R.H.E., Bhabha G., Kwaks T., Jongeneelen M., Yu W., Ophorst C., Cox F., Korse H.J., Brandenburg B. (2011). Science..

[4] Throsby M., van den Brink E., Jongeneelen M., Poon L.L.M., Alard P., Cornelissen L., Bakker A., Cox F., van Deventer E., Guan Y., Cinatl J. (2008). PLoS One..

[5] Sui J., Hwang W.C., Perez S., Wei G., Aird D., Chen L.M., Santelli E., Stec B., Cadwell G., Ali M. (2009). Nat. Struct. Mol. Biol..

[6] Wyrzucki A., Dreyfus C., Kohler I., Steck M., Wilson I.A., Hangartner L. (2014). Virology Journal.

[7] Corti D., Suguitan A.L., Pinna D., Silacci C., Fernandez-Rodriguez B.M., Vanzetta F., Santos C., Luke C.J., Torres-Velez F.J., Temperton N.J. (2010). J. Clin. Invest..

[8] de Marco D., Clementi N., Mancini N., Solforosi L., Moreno G.J., Sun X., Tumpey T.M., Gubareva L.V., Mishin V., Clementi M. (2012). PLoS One..

[9] Kashyap A.K., Steel J., Rubrum A., Estelles A., Briante R., Ilyushina N.A., Xu L., Swale R.E., Faynboym A.M., Foreman P.K. (2010). PLoS Pathog..

[10] Friesen R.H.E., Lee P.S., Stoop E.J.M., Hoffman R.M.B., Ekiert D.C., Bhabha G., Yu W., Juraszek J., Koudstaal W., Jongeneelen M. (2014). Proc. Natl. Acad. Sci. USA..

[11] Corti D., Voss J., Gamblin S.J., Codoni G., Macagno A., Jarrossay D., Vachieri S.G., Pinna D., Minola A., Vanzetta F. (2011). Science..

[12] Nakamura G., Chai N., Park S., Chiang N., Lin Z., Chiu H.., Fong R., Yan D., Kim J., Zhang J. (2013). Cell Host Microbe..

[13] Wu Y., Cho M., Shore D., Song M., Choi J., Jiang T., Deng Y.Q., Bourgeois M., Almli L., Yang H. (2015). Nat. Commun..

[14] Tharakaraman K., Subramanian V., Viswanathan K., Sloan S., Yen H.L., Barnard D.L., Leung Y.H., Szretter K.J., Koch T.J., Delaney J.C. (2015). Proc. Natl. Acad. Sci. USA..

[15] Wyrzucki A., Bianchi M., Kohler I., Steck M., Hangartner L. (2015). Virology Journal.

[16] Hu W., Chen A., Miao Y., Xia S., Ling Z., Xu K., Wang T., Xu Y., Cui J., Wu H. (2013). Virology.

[17] Li G.M., Chiu C., Wrammert J., McCausland M., Andrews S.F., Zheng N.Y., Lee J.H., Huang M., Qu X., Edupuganti S. (2012). Proc. Natl. Acad. Sci. USA..

[18] Henry Dunand C.J., Leon P.E., Kaur K., Tan G.S., Zheng N.Y., Andrews S., Huang M., Qu X., Huang Y., Salgado-Ferrer M. (2015). J. Clin. Invest..

[19] Clementi N., de Marco D., Mancini N., Solforosi L., Moreno G.J., Gubareva L.V. (2011). PLoS One..

[20] Kallewaard N.L., Corti D., Collins P.J., Neu U., McAuliffe J.M., Benjamin E., Wachter-Rosati L., Palmer-Hill F.J., Yuan A.Q., Walker P.A. (2016). Cell..

[21] Joyce M.G., Wheatley A.K., Thomas P.V., Chuang G.Y., Soto C., Bailer R.T., Druz A., Georgiev I.S., Gillespie R.A., Kanekiyo M. (2016). Cell..

[22] Dreyfus C., Laursen N.S., Kwaks T., Zuijdgeest D., Khayat R., Ekiert D.C., Lee J.H., Metlagel Z., Bujny M.V., Jongeneelen M. (2012). Science..

[23] Laursen N.S., Friesen R.H.E., Zhu X., Jongeneelen M., Blokland S., Vermond J., van Eijgen A., Tang C., van Diepen H., Obmolova G. (2018). Science..

[24] Gaiotto T., Hufton S.E. (2016). PLoS One..

[25] Hamers-Casterman C., Atarhouch T., Muyldermans S., Robinson G., Hamers C., Songa E.B., Bendahman N., Hamers R. (1993). Nature.

[26] Harmsen M.M., van Solt C.B., van Zijderveld-Van Bemmel A.M., Niewold T.A., van Zijderveld F.G. (2006). Appl. Microbiol. Biotechnol..

[27] van Heeke G., Allosery K., De Brabandere V., De Smedt T., Detalle L., de Fougerolles A. (2017). Pharmacol. Ther..

[28] Arbabi-Ghahroudi M. (2017). Front. Immunol..

[29] Jovčevska I., Muyldermans S. (2020). BioDrugs..

[30] Impagliazzo A., Milder F., Kuipers H., Wagner M.V., Zhu X., Hoffman R.M., van Meersbergen R., Huizingh J., Wanningen P., Verspuij J. (2015). Science..

[31] Arbabi Ghahroudi M., Desmyter A., Wyns L., Hamers R., Muyldermans S. (1997). FEBS Lett..

[32] Ledsgaard L., Kilstrup M., Karatt-Vellatt A., McCafferty J., Laustsen AH. (2018). Toxins (Basel)..

[33] Fischer S., Handrick R., Otte K. (2015). Biotechnol. Adv..

[34] Godakova S.A., Noskov A.N., Vinogradova I.D., Ugriumova G.A., Solovyev A.I., Esmagambetov I.B., Tukhvatulin A.I., Logunov D.Y., Naroditsky B.S., Shcheblyakov D.V. (2019). Toxins (Basel)..

[35] De Genst E., Silence K., Decanniere K., Conrath K., Loris R., Kinne J., Muyldermans S., Wyns L. (2006). Proc. Natl. Acad. Sci. USA..

[36] Stijlemans B., Conrath K., Cortez-Retamozo V., van Xong H., Wyns L., Senter P., Revets H., De Baetselier P., Muyldermans S., Magez S. (2004). J. Biol. Chem..

[37] Nobusawa E., Aoyama T., Kato H., Suzuki Y., Tateno Y., Nakajima K. (1991). Virology.

